# Novel Intronic Mutations of *TBK1* Promote Aberrant Splicing Modes in Amyotrophic Lateral Sclerosis

**DOI:** 10.3389/fnmol.2022.691534

**Published:** 2022-02-24

**Authors:** Ying-Qian Lu, Jian-Min Chen, Han Lin, Shu-Yan Feng, Chun-Hui Che, Chang-Yun Liu, Hua-Pin Huang, Zhang-Yu Zou

**Affiliations:** ^1^Department of Neurology, Fujian Medical University Union Hospital, Fuzhou, China; ^2^Institute of Clinical Neurology, Fujian Medical University, Fuzhou, China; ^3^Department of Neurophysiology, Henan Provincial People’s Hospital, Zhengzhou, China

**Keywords:** amyotrophic lateral sclerosis (ALS), TANK-binding kinase 1 (*TBK1*), targeted next-generation sequencing, intronic variants, aberrant splicing

## Abstract

TANK-binding kinase 1 (*TBK1*) has been identified as a causative gene of amyotrophic lateral sclerosis (ALS) in the Caucasian population in 2015. Here, we sequenced for *TBK1* variants in a cohort of 15 familial ALS (fALS) and 275 sporadic ALS (sALS) of Chinese origin by targeted next-generation sequencing. We identified one likely benign missense variant (p. Ser398Pro), two missense variants of uncertain significance (p. Ile37Leu and p. Tyr677Asn), and two novel heterozygous variants in introns of *TBK1*, c.1522-3T > G and c.2066 + 4A > G. We performed splicing assays through minigene plasmids and RNA pull-down assay to determine that the two substitutions of nucleotides disrupted the binding of the important splicing regulator hnRNPA1 and promoted aberrant pre-mRNA splicing modes. The c.1522-3T > G variant promoted nearly 50.0% of abnormal transcripts (3 different types of insertions and deletions (indels) in junction of intron 13-exon 14) and the c.2066 + 4A > G variant inhibited about 75.0% inclusion of exon 19, both causing premature stop codon and producing TBK1 protein without CCD2. Immunofluorescence analysis showed that the expression of *TBK1* with intronic variants was lower since less TBK1 distribution was observed in HEK293T cells. Both patients carrying *TBK1* c.1522-3T > G and c.2066 + 4A > G variants developed a rapidly progressive ALS, with a survival of 31 and 10 months, respectively. The frequency of loss of function (LoF) variants in *TBK1* was 0.73% in sALS in our cohort. We emphasize that intronic sequencing and pre-mRNA splicing analysis cannot be ignored to demonstrate the complex mutational spectrum and pathogenesis of ALS.

## Introduction

Amyotrophic lateral sclerosis (ALS) is a devastating neurological disease characterized by degeneration of upper and lower motor neurons (Chou and Norris, 1993). ALS patients are characterized by progressive muscle weakness and wasting, eventually leading to respiratory failure. Typically, patients die within 3∼5 years after symptom onset due to respiratory failure (Brown and Al-Chalabi, 2017). The incidence rate of ALS is about 2/100,000 (Rosenbohm et al., 2018).

Approximately 10% of ALS are familial ALS (fALS) and the remaining 90% are sporadic ALS (sALS) (Al-Chalabi et al., 2016). To date, mutations in more than 30 genes have been reported to contribute to the pathogenesis of ALS. The first ALS-associated gene was superoxide dismutase-1 (*SOD1*) and was described in 1993 (Rosen et al., 1993). ALS-associated mutations in *SOD1* are identified in 12–20% of fALS patients and 1–2% of sALS patients. *SOD1* is the most common gene associated with ALS in Asian populations (Zou et al., 2017). In 2008, the second contributor to ALS, TAR DNA binding protein (*TARDBP*), was discovered as an important causative gene of ALS (Gitcho et al., 2008). *TARDBP* mutations account for ∼3.3% of fALS patients and ∼0.5% of sALS (Zou et al., 2017). Fused in sarcoma (*FUS*) gene is the most common gene detected in juvenile and pediatric ALS, and the p.P525L mutation particularly tends to be associated with an aggressive and early onset form (Kwiatkowski et al., 2009; Lattante et al., 2013; Gonzalez et al., 2021). Approximately, *FUS* mutations could be found in 5% of fALS patients and 1% of sALS patients (Andersen and Al-Chalabi, 2011). A hexanucleotide (GGGGCC-) repeat expansion in the chromosome 9 open reading frame 72 (*C9orf72*) gene is the most frequent genetic cause of ALS in Caucasian populations. A *C9orf72* repeat expansion was the genetic cause in up to 35% of fALS patients and about 5% of sALS patients (Renton et al., 2011; Zou et al., 2017). Recently, many new genes have been found as causative or highly associated with ALS, like *TBK1, MATR3, CHCHD10, TUBA4A, NEK1*, and *C21orf2* (Chia et al., 2018).

*TBK*1 (TANK-binding kinase 1) gene has been identified as a causative gene of ALS in the Caucasian population in 2015 (Cirulli et al., 2015). *TBK1* is a multifunctional serine/threonine protein kinase that plays key roles in various cellular processes through phosphorylating a wide range of substrates, including neuroinflammation, innate immunity, ubiquitin-proteasome systems, cell proliferation, and autophagy pathways involving other genes also associated with ALS, such as *SQSTM1/p62, OPTN*, and *VCP* (Oakes et al., 2017; Zhao et al., 2018). TBK1 includes four domains: a serine/threonine kinase domain (KD), a ubiquitin-like domain (ULD), and two coiled-coil domains (CCD1 and CCD2) (Le Ber et al., 2015). More than 90 mutations in *TBK1*, such as non-sense mutations, frameshift, out-frame, and splice-site, have been reported to result in loss of function (LoF) of the TBK1 and lead to ALS or frontotemporal dementia (FTD) (van der Zee et al., 2017).

In this study, we aimed to screen variants of *TBK1* in a cohort of Chinese ALS patients through targeted next-generation sequencing. Moreover, we further performed *in vitro* functional experiments to address the pathogenic potential of the variants.

## Materials and Methods

### Subjects

Our study included 275 sporadic ALS (sALS) patients and 15 familial ALS (fALS) probands. The characteristics of the ALS patients were summarized in Table 1. The patients were enrolled from Fujian Medical Union Hospital and Henan Provincial People’s Hospital between January 2017 and December 2018. The diagnosis of ALS was made according to the El Escorial revised criteria (Brooks et al., 2000). Patients with clinically definite, probable, or probable laboratory-supported ALS were recruited. fALS patients were diagnosed if one or more first- or second-degree relatives developed ALS (Ludolph et al., 2015). Written informed consent was obtained from every subject to use their DNA for genetic analysis. This study was approved by the ethics committee of Fujian Medical University Union Hospital and Henan Provincial People’s Hospital. The control group included 1,000 ethnicity matched controls who had performed whole exome sequencing in AmCare Genomics Lab, Guangzhou, China.

**TABLE 1:** Characteristics of the ALS patients in this study.

	N (%)
**Gender**	
Male	173 (59.7%)
Female	117 (40.3%)
**Family history**	
Familial ALS	15 (5.2%)
Sporadic ALS	275 (94.8%)
Age of onset	55.3 ± 11.6 years
**Site of onset**	
Limbs	229 (79.0%)
Bulbar	56 (19.3%)
Respiratory	5 (1.7%)

### Genetic Analysis

Genomic DNA of each subject was extracted from peripheral blood leukocytes using a TIANamp Genomic DNA Kit (Tiangen) according to the manufacturer’s instructions. Variants in *TBK1* were screened by targeted next-generation sequencing on Illumina Hiseq sequencer (Illumina Inc., San Diego, CA). The targeted regions included all exons with intronic 50bp flanking sites of the *TBK1*, then followed by Sanger sequencing for mutation site verification on an ABI 3730 Genetic Analyzer (Foster City, CA, United States). As a result of sequencing, the mean on-target coverage was 560X with an average percentage of targets covered greater or equal to 100X of 99.8%. The identified variants were Sanger sequenced for confirmation. Patients carrying *TBK1* variants were also screened for mutations in other known ALS related genes *SOD1*, *TARDBP*, *FUS*, *VAPB*, *SPG11*, *VCP*, *PFN1*, *ANG*, *ALS2*, *DAO*, *UBQLN2*, *SIGMAR1*, *SETX*, *FIG4*, *DCTN1*, *OPTN*, *SQSTM1*, *CHCHD10*, *MATR3*, *hnRNPA1*, *hnRNPA2B1*, *ANXA11*, *KIF5A*, *TIA1*, *CCNF*, *NEK1*, *CHCHD10*, *TUBA4A*, as well as the presence of the GGGGCC expansions in the *C9orf72* gene.

### Bioinformatic Analysis

Gene variants were evaluated by their absence or frequency in the public single-nucleotide polymorphism database (dbSNP), 1,000 genome, and ExAc (Exome Aggregation Consortium). The SIFT,^1^ PolyPhen-2^2^ and Mutation Taster^3^ were used to assess the functional effects of the missense variants. The pathogenicity analysis was conducted according to the ACMG standard. The two intronic variants, c.1522-3T > G and c.2066 + 4A > G, were predicted to have changes of serine/arginine-rich splicing factors (SRSFs) using the program “ESEfinder” (version 3.0)^4^ (Cartegni et al., 2003).

### Plasmid Construction

The minigene plasmid was designed to determine the pathogenesis of c.1522-3T > G (named V-minigene 1), containing the last 146 bp of intron 12, full-length sequences from exon 13 to exon 15 (including the intron 13 and intron 14), and the first 229 bp of intron 15 of the *TBK1* gene. The corresponding wild-type minigene plasmid (the position c.1522-3 was T) was named W-minigene 1. The minigene plasmid designed to determine the pathogenesis of c.2066 + 4A > G (named V-minigene 2) contained the last 267 bp of intron 16, full-length sequences from exon 17 to exon 20 (including the intron 17, intron 18, and intron 19), and the first 350 bp of intron 20 of the *TBK1* gene; the corresponding wild type minigene plasmid (the position c.2066 + 4 was A) was named W-minigene 2. The genomic sequences were PCR amplified using a KOD-plus-Neo Kit (TOYOBO) from human DNA with primers (P1-F/R and P2-F/R for V/W-minigene 1, P3-F/R and P4-F/R for V/W-minigene 2) listed in Supplementary Table 1. Then the PCR fragments were cloned into a pCMV-cDNA-EF1a-EGFP-BGH-expressing plasmid using a ClonExpress MultiS One Step Cloning Kit (Vazyme Biotech). The DNA sequence of FLAG was cloned and added in the front of the *TBK1* gene in the minigene plasmids for immunofluorescence analysis. The identity of all the minigene plasmids was verified by Sanger sequencing. T-A cloning plasmids were constructed using a pMD™ 19-T Vector Kit (Takara).

### Cell Culture and Transfection

The HEK293T and HeLa cell lines were cultured in Dulbecco’s modified Eagle’s Medium (DMEM) (Gibco) containing 10% fetal bovine serum (FBS) (Gibco) and 1% penicillin/streptomycin (PS) (Thermo Fisher Scientific). Cells were dissociated using 0.05% Trypsin/EDTA (Gibco) and were cultured in a 37°C incubator within 5% CO_2_ atmosphere. All of the plasmids were transfected using Lipofectamine 3000 Reagent (Thermo Fisher Scientific) according to the standard instructions.

### RNA Extraction and Splicing Assays

**FIGURE 1:**
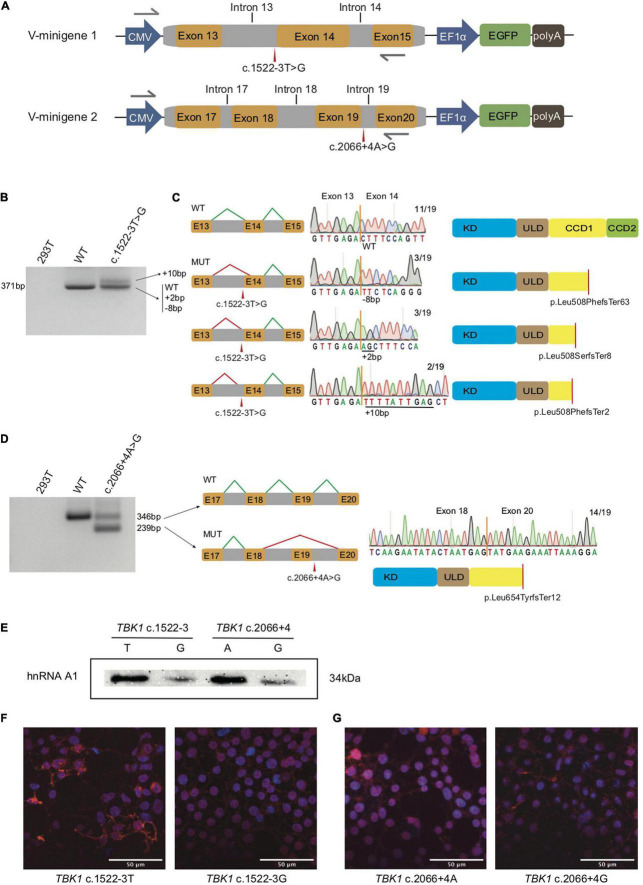
Splicing modes verification and functional characterization of the two *TBK1* intronic variants. **(A)** Construction of V-minigene 1 (bearing *TBK1* c.1522-3T > G variant) and V-minigene 2 (bearing *TBK1* c.2066 + 4A > G variant) plasmids. The minigene plasmids contain CMV promotor, consecutive genomic DNA, EF1α promotor and EGFP sequences. Yellow boxes indicate exons, gray boxes indicate introns, gray arrows indicate primers. **(B)** Agarose gel electrophoresis of RT-PCR products, lane 1: cDNA from HEK293T cells (blank control), lane 2: cDNA from W-minigene 1 plasmid (wild type control, bearing *TBK1* c.1522-3T), lane 3: cDNA from V-minigene 1 plasmid (bearing *TBK1* c.1522-3T > G variant). **(C)** Left: schematics of normal splicing mode (WT) and aberrant splicing modes (MUT) of V-minigene 1 plasmid; yellow boxes indicate exons, gray boxes indicate introns, green lines show normal splicing modes, red lines show aberrant splicing modes, E13: exon 13, E14: exon 14, E15: exon 15. Middle: T-A clone results of RT-PCR products from V-minigene 1 plasmid. Right: Schematics of TBK1 protein corresponding to the former splicing modes; red lines indicate positions of premature stop codon. **(D)** Left: agarose gel electrophoresis of RT-PCR products, lane 1: cDNA from HEK293T cells (blank control), lane 2: cDNA from W-minigene 2 plasmid (wild type control, bearing *TBK1* c.2066 + 4A), lane 3: cDNA from V-minigene 2 plasmid (*TBK1* c.2066 + 4A > G variant). Middle: schematic of normal splicing mode (WT) and aberrant splicing mode (MUT) of V-minigene 2 plasmid; green lines show normal splicing modes, red lines show aberrant splicing modes, yellow boxes indicate exons, gray boxes indicate introns, E17: exon 17, E18: exon 18, E19: exon 19, E20: exon 20. Right: T-A clone results of RT-PCR products from V-minigene 2 plasmid and schematic of TBK1 protein corresponding to the MUT splicing mode. **(E)** RNA pull-down assay and western blot analysis. Synthetic RNAs were labeled with biotin and incubated with nuclear extract from HeLa cells, then the RNA-binding protein complexes were interacted with anti-hnRNPA1 antibody specifically and separated by SDS-PAGE. **(F,G)** Immunofluorescence analysis of subcellular distribution of TBK1 in HEK293T cells overexpressed by TBK1 cDNA carrying c.1522-3T or c.1522-3G **(F)** and TBK1 cDNA carrying c.2066 + 4A or c.2066 + 4G **(G)** was visualized by anti-FLAG antibody followed by anti-mouse Alexa Fluor 594 (red)-conjugated secondary antibody and DAPI (blue) staining. Scale bar: 50 μm.

Total RNA of HEK293T and HeLa cells transfected with minigene plasmids was extracted 2 days later using TRIzol reagent (Invitrogen) following the manufacturer’s recommendations. For cDNA synthesis, we used 1 μg of each RNA sample per 20 μl reverse transcription reaction using a HiScript II Q RT SuperMix Kit (Vazyme Biotech) according to the manufacturer’s instructions. RT-PCR was performed using a KOD-plus-Neo Kit (TOYOBO) with primers (RT1-F/R for V/W-minigene 1 and RT2-F/R for V/W-minigene 2) listed in Supplementary Table 1 (the upstream primers RT1/2-F were the same). To distinguish the pre-mRNA splicing isoforms originating from HEK293T (or HeLa) cells and minigene plasmids, the upstream primers for RT-PCR were designed specifically to bind to the back-bone of minigene plasmids and would not bind to the sequences of HEK293T (or HeLa) cells (Figure 1A). Then Sanger sequencing was performed to determine the detailed pre-mRNA splicing modes using the upstream primers. The splicing assays were repeated three times independently.

### RNA Pull-Down Assay

RNA pull-down assay was performed with a Pierce™ Magnetic RNA-Protein Pull-Down Kit (Thermo Fisher Scientific) according to the manufacturer’s instructions. RNA TBK1 r.1522-3U (5′-UUUGGUUUUAUUUAGCUUUCCAGUU-3′), TBK1 r.1522-3G (5′- UUUGGUUUUAUUGAGCUUUCCAGUU-3′), TBK1 r. 2066 + 4A (5′- GACUCUUGGGUAAGAAACUCAUC AU-3′), and TBK1 r. 2066 + 4G (5′- GACUCUUGGGUAG GAAACUCAUCAU-3′) were synthesized by Sangon Biotech and were biotin labeled at 3′ End using a Pierce RNA 3′ End Desthiobiotinylation Kit (Thermo Fisher Scientific). The biotin-labeled RNAs were added to streptavidin magnetic beads and incubated for 1 h at 4°C with agitation. After washing, HeLa nuclear extract was added to the RNA-beads mixture and incubated for 2 h at 4°C with agitation. RNA-binding protein complexes were analyzed by SDS-PAGE and immunoblotting with anti-hnRNPA1 antibody (Abcam). The experiments were repeated three times independently.

### Immunofluorescence Analysis

HEK293T cells cultured on glass coverslips were fixed with 4% PFA diluted in PBS for 30 min at room temperature and then incubated with primary anti-FLAG antibody (1:1,000, Sigma) followed by Alexa Fluor 594-conjugated anti-mouse secondary antibody (1:500, Sigma). The primary and secondary antibodies were both diluted in PBS containing 1% BSA and 0.3% Tx-100. At last, samples were briefly stained with DAPI (1:2,500, Sigma). Images were captured with Leica TCS SP8 confocal microscopy.

## Results

### Identification of *TBK1* Variants

**FIGURE 2:**
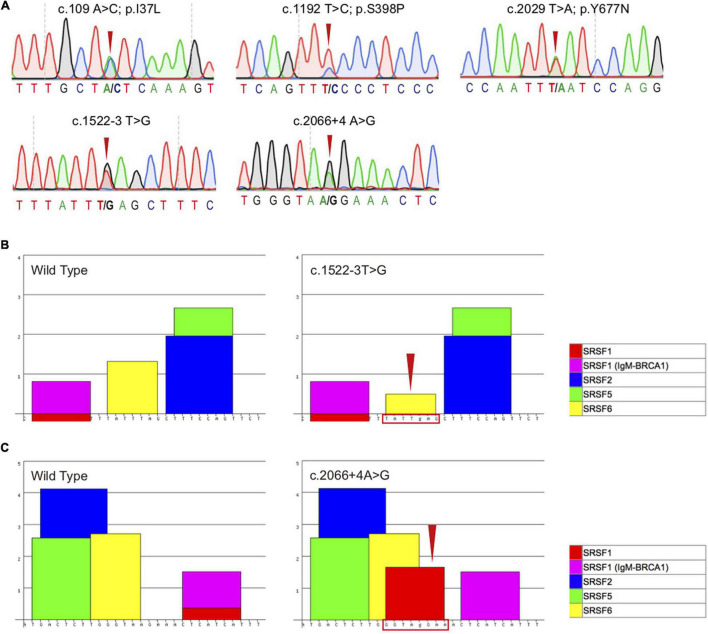
Sequencing chromatogram and bioinformatics analysis of the *TBK1* variants. **(A)** Sequencing chromatograms of the five *TBK1* variants (c.109A > C, c.1192T > C, c.2029T > A, c.1522-3T > G and c.2066 + 4A > G) detected in this study. **(B)** Bioinformatics analysis of the wild type sequences and mutated sequences with the *TBK1* c.1522-3T > G variant using ESEfinder 3.0, the program recognized that the TATTTAG (the binding site of SRSF6) (left) transformed to TATTgAG (right, indicated by red arrow and frame). **(C)** Bioinformatics analysis of the wild type sequences and mutated sequences with the *TBK1* c.2066 + 4A > G variant using ESEfinder 3.0, the program recognized that a new motif GGTAgGAA (the binding site of SRSF1) was generated (right, indicated by red arrow and frame).

Three heterozygous missense variants, c.109A > C (p.Ile37Leu), c.1192T > C (p.Ser398Pro), and c.2029T > A (p.Tyr677Asn) in the exons of *TBK1* gene (Figure 2A and Table 2), were each identified in one sALS patient. The p.Ser398Pro and p.Tyr677Asn variants had been reported in dbSNP (rs781434264 and rs1163013930), while the p.Ile37Leu variant was novel. Two novel heterozygous variants, c.1522-3T > G and c.2066 + 4A > G, located in intron 13 and intron 19 of *TBK1*, respectively, were each identified in one sALS case (Figure 2A and Table 2). No *TBK1* variants were found in the fALS patients. No variants of other ALS-related genes were identified in the five sALS cases. No parental DNA samples of these patients were available for further analysis. All five TBK1 variants were absent from the population database 1000genome and ExAC, ExAC, as well as 1,000 ethnicity matched controls. The novel variant p. Ile37Leu was predicted to be “damaging” and “benign” with SIFT and Polyphen-2, respectively; and the variant was predicted to be “disease causing” with Mutation Taster. The p. Ser398Pro and p.Tyr677Asn variants were both predicted to be “tolerable” with SIFT and “benign” with Polyphen-2. With Mutation Taster, the p. Ser398Pro and p. Tyr677Asn variants were predicted to be “polymorphism” and “disease causing,” respectively. The p. Ile37Leu and p. Tyr677Asn variants were interpreted as variants of uncertain significance (VUS) while the p.Ser398Pro variant was likely benign according to the 2015 American College of Medical Genetics and Genomics (ACMG) guidelines (Table 2).

**TABLE 2:** Genetic profile of the *TBK1* variants identified in this study.

Location	Nucleoid changes	Amino acids changes	Protein domain	dbSNP/novel	1,000 genome	ExAC	Evolutionary conservation	SIFT score	PolyPhen-2	Mutation Taster	ACMG
Exon 3	c.109A > C	p. Ile37Leu	KD	Novel	0	0	No	0.008 (damaging)	0.006 (benign)	0.797 (disease causing)	VUS
Exon 10	c.1192T > C	p. Ser398Pro	ULD-CCD1	rs781434264	0	0	No	0.15 (tolerable)	0.261 (benign)	0.549 (polymorphism)	Likely benign
Exon 19	c.2029T > A	p. Tyr677Asn	CCD2	rs1163013930	0	0	Yes	0.279 (tolerable)	0.084 (benign)	1.0 (disease causing)	VUS
Intron 13	c.1522-3T > G	p.Leu508PhefsTer63/ p.Leu508SerfsTer8 /p.Leu508PhefsTer2	-	Novel	-	-	-	-	-		-
Intron 19	c.2066 + 4A > G	p.Leu654TyrfsTer12	-	Novel	-	-	-	-	-	-	-

*VUS, Variant of uncertain significance.*

### Prediction Pathogenicity by Bioinformatics Analysis

As adjacent to the splicing sites, both *TBK1* c.1522-3T > G and c.2066 + 4A > G variants were likely to be pathogenic based on the potential interference in the splicing regulator. To determine the pathogenic significance, we conducted bioinformatics analysis of the variants using the ESEfinder 3.0. The ESEfinder 3.0 predicted that the c.1522-3T > G variant transformed the serine/arginine-rich splicing factor 6 (SRSF6) (TATTTAG termed to TATTgAG) and the c.2066 + 4A > G variant generated a new SRSF1 (GGTAgGAA) (Figures 2B,C). It thus appeared that the preexisting splicing modes might be interfered.

### Splicing Modes Verification by Minigene Plasmids

In the light of the prediction, we performed *in vitro* splicing assays using minigene plasmids harboring wild type sequence (named W-minigene 1 and W-minigene 2) or the intronic variants (named V-minigene 1 and V-minigene 2) of *TBK1* to mimic the endogenous splicing processes (Figure 1A). These minigene plasmids were transfected into HEK293T cells, then total RNA was extracted and reverse transcription followed by PCR (RT-PCR) was performed to assess the splicing modes following a previous study (Lin et al., 2020). We employed TA clone and Sanger sequencing to confirm the detailed splicing modes.

There was more than one kind of RT-PCR product from the cDNA of V-minigene 1 plasmid (Figure 1B). In addition to the wild type splicing mode (termed WT), Sanger sequencing revealed that the minigene plasmid harboring c.1522-3T > G variant (V-minigene 1) promoted another 3 types of aberrant splicing modes (termed MUT) (Figure 1C). As the results of TA clone, 3 (15.79%) showed deletion of 8 nucleotides, 3 (15.79%) showed insertion of 2 nucleotides, and 2 (10.53%) showed insertion of 10 nucleotides in all the 19 clones (Figure 1C). The aberrant splicing modes all caused premature stop codon (p. Leu508PhefsTer63, p. Leu508SerfsTer8, and p. Leu508PhefsTer2) of TBK1 protein (Figure 1C). The minigene plasmid bearing c.2066 + 4A > G variant (V-minigene 2) promoted 2 types of splicing modes, one was the wild type splicing mode (termed WT) and the other one was skipping of exon 19 (termed MUT). The splicing mode also caused premature stop codon (p. Leu654TyrfsTer12) of TBK1 protein (Figure 1D). TA clone and Sanger sequencing results revealed that 14/19 (73.68%) of clones showed exclusion of exon 19 (Figure 1D). To ascertain whether splicing modes of TBK1 could be interfered with by different conditions of cells, we repeated the *in vitro* splicing assays three times at different times independently in HEK293T cells. Splicing assays were also repeated three times in HeLa cells to avoid the interference of different cell types in splicing modes. The TA clone and Sanger sequencing results showed that splicing modes of TBK1 had no changes in different cell conditions or types (Supplementary Figure 1).

### Functional Characterization of the Two *TBK1* Intronic Variants

We performed an RNA pull-down assay to confirm changes of the SRSF1 and SRSF6 binding at the sequences around the two *TBK1* intronic variants. Biotin-labeled RNAs were incubated with HeLa nuclear extract to form RNA-binding protein complexes. Then, the presence of SRSF1 or SRSF6 in the pull-down materials was assessed by western blot analysis. As a result, the SRSF1 and SRSF6 binding at the sequences around the intronic variants had no significant differences with the binding at the reference sequences (data did not show). We considered that this may be the transformation or generating of SRSF1/SRSF6 was predicted in the website, which is just indicative of the possibility of aberrant splicing. We then identified the hnRNPA1 binding at the region around the two *TBK1* variants. The hnRNPA1 was one of the key splicing regulators bound at the splicing sites usually. Compared to the wild type sequences (c.1522-3U and c.2066 + 4A), RNAs bearing the variants (c.1522-3G and c.2066 + 4G) showed obviously lower binding of hnRNPA1 (Figure 1E). These findings indicated that hnRNPA1 binding at the sequences around the intronic variants was disrupted with the generation of new negative splicing-regulatory elements possibly, which promotes aberrant splicing. We also performed immunofluorescence analysis showing the subcellular distribution of TBK1 (overexpressed by TBK1 cDNA with c.1522-3T/G and TBK1 cDNA with c.2066 + 4A/G) visualized by anti-FLAG antibody in HEK293T cells. HEK293T cells overexpressed with TBK1 cDNA bearing variants showed less TBK1 distribution both in the nucleus and cytoplasm than the cells overexpressed with wild type TBK1 cDNA (Figures 1F,G). The results indicated that the truncated TBK1 protein produced by *TBK1* intronic variants had a lower level of expression than the wild type TBK1 protein.

### Clinical Features of the Patients Carrying *TBK1* Intronic Variants

Patient 1 (bearing *TBK1* c.1522-3T > G variant) was a 71-year-old woman referred due to dysarthria for 6 months and weakness of left hand for 2 months. Neurological examination revealed severe dysarthria, obvious atrophy of the tongue with fibrillation. Muscle strength was grade 4 in muscles of the left hand and grade 5 in other limbs. Fasciculations were observed in the left upper limb. Deep tendon reflexes were increased in the left upper limb. Palm-chin reflex was elicited bilaterally. She scored 43/48 on ALS Functional Rating Scale-Revised (ALSFRS-R) and 28/30 on Montreal Cognitive Assessment testing. Electromyography demonstrated acute denervation in all limbs and thoracic muscles, as well as chronic reinnervation in all limbs and sternocleidomastoid muscles. Laboratory testing for diabetes, vasculitis, and anti-double-stranded DNA, anti-Ro/SSA, anti-La/SSB, anti-nuclear antibodies, and anti-neuronal antibodies were negative. Brain MRI showed an increased symmetrical fluid attenuation inversion recovery (FLAIR) signal intensity in the posterior limb of the internal capsule. One year after onset, she developed dysphagia and weakness of the right hand. She began to have difficulties in walking and had a weight loss of 10 kilograms due to severe dysphagia 2 years after onset. She refused to perform percutaneous endoscopic gastrostomy and died 31 months after onset.

Patient 2 (bearing *TBK1* c.2066 + 4A > G variant) was a 60-year-old man who presented with weakness of the left upper and lower limbs at the age of 59 years. The symptom progressed quickly to involve the right arm 5 months later. The patient had some difficulties in climbing upstairs and began to develop dysarthria and slight choking when eating. On examination 6 months after disease onset, obvious atrophy of muscles of upper and lower limbs and the tongue with fasciculations were noticed. Deep tendon reflexes were increased in all limbs but Babinski’s sign was not elicited. He was cognitively normal. The initial assessment of the ALSFRS-R score was 34/48 with an estimated progression rate of 2.3 points/month since symptom onset. Electromyography demonstrated acute and chronic neurogenic changes in the upper limb, lower limbs thoracic, and sternocleidomastoid muscles. Laboratory testing for diabetes, vasculitis, anti-nuclear antibodies, and anti-neuronal antibodies were negative. Brain MRI was normal. The patient refused to use percutaneous endoscopic gastrostomy or nasogastric tube feeding and died due to asphyxia caused by choking on food10 months after onset.

## Discussion

In this study, we identified five *TBK1* variants by targeted next-generation sequencing in a Chinese cohort of 275 sALS patients and 15 fALS probands, including three missense variants (p.Ile37Leu, p.Ser398Pro, p.Tyr677Asn) and two novel intronic mutations (c.1522-3T > G, c.2066 + 4A > G). Online *in silico* programs were used to predict the potential impacts of the three missense variants on the structure and function of TBK1 protein with SIFT, Polyphen-2, and Mutation Taster. The p.Ile37Leu and p.Tyr677Asn were VUS while c.1192T > C (p. Ser398Pro) was a likely benign missense variant according to ACMG guidelines. However, the results of *in silico* programs should be interpreted with cautions and further experiments will need to determine the pathogenicity of these missense TBK1 variants.

The two novel intronic mutations (c.1522-3T > G, c.2066 + 4A > G) did not affect the function of TBK1 protein *via* amino-acid substitution. Instead, they interfered with normal pre-mRNA splicing due to disruption of the hnRNPA1 binding and forming aberrant splicing modes. Our results demonstrated that the c.1522-3T > G mutation promoted nearly 50% of abnormal transcripts with 3 different types of insertions and deletions (indels) in the junction of intron 13-exon 14 and the c.2066 + 4A > G mutation inhibited ∼75.0% inclusion of exon 19. The aberrant splicing modes caused premature stop codon and shifted the codon triplets of the genetic codes of mRNA, resulting in truncated protein without CCD2. Protein-truncated mutations in *TBK1* caused the reduction of TBK1 transcripts and/or protein levels, which was an indication of pathogenicity (Pottier et al., 2015). We showed that the expression level of *TBK1* with intronic mutations was lower than the wild type *TBK1* through immunofluorescence analysis. CCD2 of TBK1 is involved in binding to and phosphorylating OPTN, which is an important regulator of autophagy (Swift et al., 2021). Previous research showed that altering the TBK1 downstream regulatory pathways might be the pathogenic mechanism of most *TBK1* mutations (Freischmidt et al., 2015; Chia et al., 2018). Some *TBK1* mutations may contribute to disease pathogenesis by disrupting mitophagic flux or inducing mitochondrial stress, preventing mitophagy components from performing cellular roles. Moreover, deficient mitophagy might stimulate innate immune pathways and promote the accumulation of toxic aggregates (Harding et al., 2021). Another research suggested that TBK1 haploinsufficiency might contribute to ALS/FTD through vesicular trafficking, a new molecular pathway different from previous thought (Lu et al., 2021). The pathogenic mechanisms above could explain the pathogenicity of these two intronic mutations in the two ALS patients.

In this study, the frequency of variants and LoF variants in *TBK1* in sALS was 1.82% (5/275) and 0.73% (2/275), respectively. In previous studies in the Chinese population, the frequency of LoF *TBK1* mutations in sporadic ALS ± FTD was between 0 and 0.62% (none in 271 cases, one out of 162 patients, one out of 207 patients, and one out of 608 cases, respectively) (Liu et al., 2021). Combined with the results of our cohort, the frequency of LoF mutations in sporadic ALS ± FTD was 0.38% (5/1,323) in the Chinese population. Only one *TBK1* LoF mutation was identified in familial ALS ± FTD in the Chinese population (0.77%, 1/130) (Liu et al., 2021). The frequency of *TBK1* LoF mutations in sporadic ALS ± FTD in the Chinese population is similar to the frequency of the Japanese population (0.42%, 3/713), lower than the frequency of the Korean population (0.78%, 1/129) and the European populations (1.7%, 11/665) (van der Zee et al., 2017). The frequency of *TBK1* LoF mutations in fALS in the Chinese population is lower than the frequency of the European population (1.0%, 7/699) (de Majo et al., 2018).

The reported clinical features of ALS associated with *TBK1* mutations are variable in the age of onset, progression of the disease, survival duration, and presence of cognitive impairment and extrapyramidal symptoms (Van Mossevelde et al., 2016). The ALS patients carrying *TBK1* intronic mutations in the study showed typical clinical features of ALS without cognitive impairment. Their symptoms progressed relatively quickly and the survival duration was only 31 and 10 months, respectively. We supposed that both patients with intronic mutations manifesting severe clinical symptoms may be attributed to the indels in junction of intron-exon or the skipping of exons result from aberrant splicing modes, which produced TBK1 protein containing truncated CCD1 without CCD2. Besides, the patient with *TBK1* c.2066 + 4A > G mutation showed more severe clinical features than the one with c.1522-3T > G mutation may be due to the higher proportion of the abnormal TBK1 transcripts.

In summary, we identified one likely benign missense variant, two missense VUS, and two novel intronic mutations of *TBK1* in a Chinese ALS cohort. Combined with other studies in the Chinese population, the frequencies of LoF mutations in sporadic ALS ± FTD and familial ALS ± FTD were 0.38% and 0.77%, respectively. The pathogenic mechanisms of the two novel mutations were considered as disruption of the interaction between TBK1/OPTN due to truncated TBK1 protein result from aberrant splicing and TBK1 haploinsufficiency. Several other molecular pathways might also involve in the pathogenicity of the TBK1 mutations. We confirmed that substitution of a single nucleotide in the splicing regulators can result in partial or full-length exon or intronic sequence inclusion or exclusion, generating aberrant splicing and producing truncated protein. The findings also emphasize the importance of intronic sequencing in detecting variants and the need to combine with an analysis of pre-mRNA splicing modes in determining the pathogenic mechanisms of ALS. ASOs or gene-editing technology such as CRISPR/Cas9 may be potential treatment strategies worthy for further explorations by masking or disrupting the negative splicing-regulatory elements to correct the aberrant splicing (Jin et al., 2020; Li et al., 2020).

## Data Availability Statement

The data presented in the study can be found in the link below: https://www.ncbi.nlm.nih.gov/sra/PRJNA750742.

## Ethics Statement

The studies involving human participants were reviewed and approved by the Fujian Medical University Union Hospital and Henan Provincial People’s Hospital. The patients/participants provided their written informed consent to participate in this study. The animal study was reviewed and approved by Fujian Medical University Union Hospital and Henan Provincial People’s Hospital. Written informed consent was obtained from the individual(s) for the publication of any potentially identifiable images or data included in this article.

## Author Contributions

Z-YZ and H-PH designed and conceived the study. Z-YZ and Y-QL performed the analysis of mutations in all the patients. Y-QL and J-MC performed the functional experiments and analysis of data. Y-QL wrote the manuscript. Z-YZ critically revised the manuscript. All remaining authors participated in analysis of data, discussion of the final manuscript.

## Conflict of Interest

The authors declare that the research was conducted in the absence of any commercial or financial relationships that could be construed as a potential conflict of interest.

## Publisher’s Note

All claims expressed in this article are solely those of the authors and do not necessarily represent those of their affiliated organizations, or those of the publisher, the editors and the reviewers. Any product that may be evaluated in this article, or claim that may be made by its manufacturer, is not guaranteed or endorsed by the publisher.

## Footnotes

1
https://sift.bii.a-star.edu.sg

2
http://genetics.bwh.harvard.edu/pph2

3
http://www.mutationtaster.org

4
http://krainer01.cshl.edu/cgi-bin/tools/ESE3/esefinder.cgi

## Supplementary Material

Supplementary Figure 1 Splicing modes of *TBK1* with intronic variants in HEK293T and HeLa cells. **(A)** T-A clone results of normal splicing mode (WT) and three aberrant splicing modes (deletion of 8bp, insertion of 2bp, and insertion of 10bp) of *TBK1* with c.1522-3T > G in HEK293T and HeLa cells, respectively. Fractions below roman numerals I, II, and III showed the proportion of the four splicing modes (WT and aberrant) respectively, from three independent repetitions. **(B)** Schematic of normal splicing mode (WT) and aberrant splicing mode (MUT) of *TBK1* with c.2066 + 4A > G in HEK293T and HeLa cells, respectively. Green lines show normal splicing modes, red lines show aberrant splicing modes, yellow boxes indicate exons, gray boxes indicate introns. E17: exon 17, E18: exon 18, E19: exon 19, E20: exon 20. Fractions below roman numerals I, II, and III showed the proportion of the two splicing modes (WT and MUT) respectively, from three independent repetitions.
