# Supplementary figures and images for: Novel Intronic Mutations of TBK1 Promote Aberrant Splicing Modes in Amyotrophic Lateral Sclerosis
Source: Front Mol Neurosci. 2022 Feb 24;15:691534. doi: 10.3389/fnmol.2022.691534 (PMC8908445; doi:10.3389/fnmol.2022.691534)

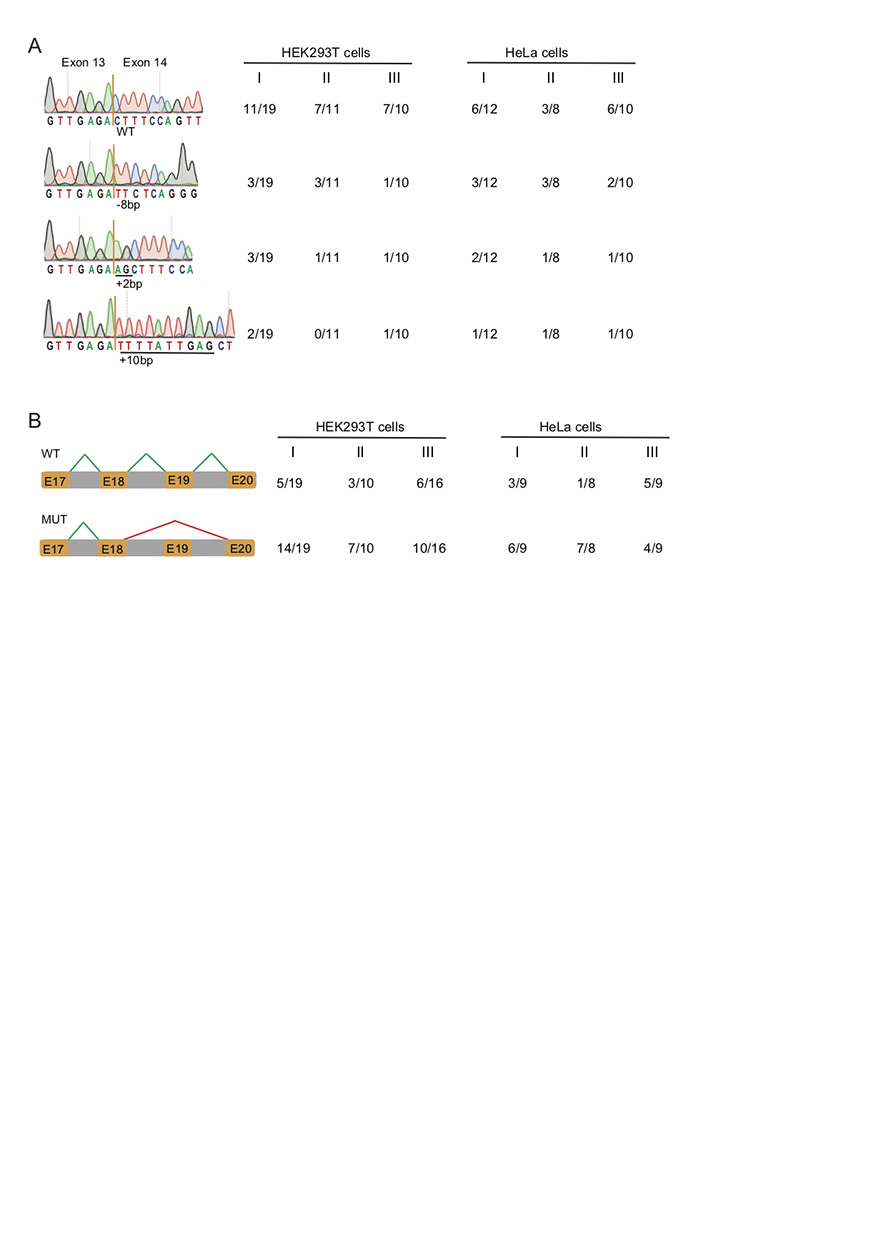

Supplement: Supplementary Figure 1 — Splicing modes of TBK1 with intronic variants in HEK293T and HeLa cells. (A) T-A clone results of normal splicing mode (WT) and three aberrant splicing modes (deletion of 8bp, insertion of 2bp, and insertion of 10bp) of TBK1 with c.1522-3T > G in HEK293T and HeLa cells, respectively. Fractions below roman numerals I, II, and III showed the proportion of the four splicing modes (WT and aberrant) respectively, from three independent repetitions. (B) Schematic of normal splicing mode (WT) and aberrant splicing mode (MUT) of TBK1 with c.2066 + 4A > G in HEK293T and HeLa cells, respectively. Green lines show normal splicing modes, red lines show aberrant splicing modes, yellow boxes indicate exons, gray boxes indicate introns. E17: exon 17, E18: exon 18, E19: exon 19, E20: exon 20. Fractions below roman numerals I, II, and III showed the proportion of the two splicing modes (WT and MUT) respectively, from three independent repetitions. [file Image_1.JPEG]
